# Preoperative Glycosylated Haemoglobin Screening to Identify Older Adult Patients with Undiagnosed Diabetes Mellitus—A Retrospective Cohort Study

**DOI:** 10.3390/jpm14020219

**Published:** 2024-02-19

**Authors:** Robert van Wilpe, Mark L. van Zuylen, Jeroen Hermanides, J. Hans DeVries, Benedikt Preckel, Abraham H. Hulst

**Affiliations:** 1 Department of Anaesthesiology, Amsterdam University Medical Center, University of Amsterdam, Meibergdreef 9, Postbus 22660, 1105 AZ Amsterdam, The Netherlands; r.vanwilpe@amsterdamumc.nl (R.v.W.); m.l.zuylenvan@amsterdamumc.nl (M.L.v.Z.); j.hermanides@amsterdamumc.nl (J.H.); a.h.hulst@amsterdamumc.nl (A.H.H.); 2 Department of Paediatric Intensive Care, Amsterdam University Medical Center, University of Amsterdam, Meibergdreef 9, Postbus 22660, 1105 AZ Amsterdam, The Netherlands; 3 Department of Endocrinology, Amsterdam University Medical Center, University of Amsterdam, Meibergdreef 9, Postbus 22660, 1105 AZ Amsterdam, The Netherlands

**Keywords:** HbA1c, preoperative, undiagnosed diabetes

## Abstract

More than 25% of older adults in Europe have diabetes mellitus. It is estimated that 45% of patients with diabetes are currently undiagnosed, which is a known risk factor for perioperative morbidity. We investigated whether routine HbA1c screening in older adult patients undergoing surgery would identify patients with undiagnosed diabetes. We included patients aged ≥65 years without a diagnosis of diabetes who visited the preoperative assessment clinic at the Amsterdam University Medical Center and underwent HbA1c screening within three months before surgery. Patients undergoing cardiac surgery were excluded. We assessed the prevalence of undiagnosed diabetes (defined as HbA1c ≥ 48 mmol·mol^−1^) and prediabetes (HbA1c 39–47 mmol·mol^−1^). Using a multivariate regression model, we analysed the ability of HbA1c to predict days alive and at home within 30 days after surgery. From January to December 2019, we screened 2015 patients ≥65 years at our clinic. Of these, 697 patients without a diagnosis of diabetes underwent HbA1c screening. The prevalence of undiagnosed diabetes and prediabetes was 3.7% (95%CI 2.5–5.4%) and 42.9% (95%CI 39.2–46.7%), respectively. Preoperative HbA1c was not associated with days alive and at home within 30 days after surgery. In conclusion, we identified a small number of patients with undiagnosed diabetes and a high prevalence of prediabetes based on preoperative HbA1c screening in a cohort of older adults undergoing non-cardiac surgery. The relevance of prediabetes in the perioperative setting is unclear. Screening for HbA1c in older adult patients undergoing non-cardiac surgery does not appear to help predict postoperative outcome.

## 1. Introduction

Diabetes mellitus (DM) is one of the most prevalent comorbidities in surgical patients and is associated with perioperative morbidity [1,2,3,4,5]. Perioperative care providers can take measures to prevent perioperative dysglycaemia in patients with DM, e.g., by preoperative dose adjustments for diabetes medications and regular blood glucose monitoring. However, DM may not always be diagnosed at the time of surgery, as the lack of symptoms during the early course of type 2 DM can delay its diagnosis considerably [6]. Extrapolations from population-based studies indicate that over 30% of patients with DM between the ages of 20 and 79 years living in high-income countries in Europe are currently undiagnosed [7]. Since the risk of developing DM increases with age, it is likely that a substantial number of older adult patients visiting the preoperative assessment clinic have yet undiagnosed DM. Identifying these patients is relevant, because hyperglycaemia is associated with a worse in-hospital outcome in patients with undiagnosed DM, compared to those with known DM [8,9,10,11].

The diagnosis of DM is based on criteria for plasma glucose or glycosylated haemoglobin (HbA1c) [12,13,14]. Plasma glucose criteria include the fasting plasma glucose (FPG) value and the two-hour plasma glucose value during an oral glucose tolerance test (OGTT), which may not be feasible at the time of preoperative screening, as both tests require at least 8 h of prior fasting. Formed by the glycation of the haemoglobin protein, HbA1c reflects the average glycaemic control over the last three months (i.e., the average life span of red blood cells) and is a predictor of both perioperative glucose control and adverse outcome after surgery [2,15,16,17,18,19], even within the prediabetes range (39–47 mmol·mol^−1^) [15]. Although the preoperative measurement of HbA1c in patients with DM is recommended in several perioperative guidelines [20,21,22], it is not commonly used as a screening tool for DM during preoperative assessment.

In Europe, the estimated prevalence of DM in people over 65 years of age is 27.8% [23]. We hypothesised that preoperative screening for DM by the routine measurement of HbA1c in older adult patients would identify patients with undiagnosed DM. We also aimed to evaluate the relationship between HbA1c and postoperative outcome, measured as days alive and at home within 30 days after surgery (DAH30), in patients without a history of DM.

## 2. Materials and Methods

### 2.1. Design and Setting

This retrospective observational cohort study was conducted at the Amsterdam UMC—location AMC, a tertiary teaching hospital. The study has been approved by the medical ethical committee AMC (decision W19_044 #19.067). Reporting is in accordance with the STROBE statement on the reporting of observational studies [24]. 

### 2.2. Participants

We identified all patients aged ≥65 years who visited the preoperative assessment clinic of the Amsterdam UMC—location AMC from January to December 2019. During this period, an HbA1c measurement was to be routinely performed as part of the preoperative assessment in patients aged ≥65 years who planned to undergo non-emergency procedures requiring anaesthetic care, irrespective of a previous diagnosis of DM. Exceptions included patients with telephone or video consultations and inpatients. Patients with a history of DM and those who objected to the collection of data for research purposes were excluded from this study. Cardiac surgery patients were excluded because their inclusion could introduce bias with regard to the DAH30 outcome. Patients with an HbA1c value measured within 3 months before surgery were included in the primary analysis. In case a patient visited the preoperative assessment clinic more than once during the study period due to multiple surgeries (referred to as duplicates), only the first visit’s HbA1c value was included into the analysis. The management in case of an elevated HbA1c was left to the discretion of the attending physician at the preoperative assessment clinic.

### 2.3. Outcome Parameters

The main outcome was the prevalence of undiagnosed DM, defined as an HbA1c ≥ 48 mmol·mol^−1^ (>6.5%) in patients without a diagnosis of DM [12,13]. The prevalence of prediabetes was also assessed, defined as an HbA1c between 39 and 47 mmol·mol^−1^ (5.7–6.5%) as per the American Diabetes Association (ADA) diagnostic criteria [12]. DAH30 was used as a measure of postoperative outcome [25]. This outcome combines length of stay (LOS), readmission and mortality into a single patient-centred measure and is considered more reliable than the retrospective assessment of postoperative complications alone [25]. DAH30 has been shown to have construct validity and is associated with postoperative complications [25,26]. Hospitalisation days within 30 days after surgery were calculated by adding the LOS after index surgery (day 0) to the number of readmission days within this time period. DAH30 was acquired by subtracting these hospitalisation days from 30. In case of death within 30 days, the DAH30 was scored as zero. 

### 2.4. Data Sources

Patient data were collected by manual review of the electronic health records. In order to minimise the risk of human error, all cases of undiagnosed DM were confirmed by chart review by a second investigator. Demographic characteristics collected included age, gender and body mass index (BMI). Medication use and coexisting cardiovascular disease were assessed. Within the electronic health records, relevant keywords were searched to minimise the risk of errors. The American Society of Anesthesiologists Physical Status (ASA) category was based on the judgment of the attending anaesthetist who screened the patient. Patients classified as ASA ≥ 3 have a severe systemic disease which is either not life-threatening (ASA 3) or a constant threat to life (ASA 4). Functional capacity was based on the patient-reported maximum metabolic equivalent of task (MET) level achievable. Surgical speciality and the type of anaesthesia were recorded. Finally, surgical risk was rated on a 3-point scale—low, intermediate or high risk—in accordance with the European Society of Cardiology (ESC) and the European Society of Anaesthesiology (ESA) guidelines on non-cardiac surgery [22]. 

### 2.5. Statistical Methods

All analyses were performed using SPSS version 26 (IBM, Armonk, NY, USA). Normality was assessed using Q-Q plots and the Shapiro–Wilk test. Based on previous data in the general preoperative population, 4–7% of patients have undiagnosed DM [2,27,28]. In older adults, we expected this to be at least 10%. To detect this proportion with a 95% confidence level that extends 2.5% both ways, we needed a sample size of at least 554 patients [29]. The binomial confidence intervals of prevalences were calculated using the Clopper–Pearson exact method [30]. Binary logistic regression analysis was performed to identify predictors of an elevated HbA1c (i.e., ≥39 mmol·mol^−1^). Quantile regression was used to analyse the DAH30 outcome because its distribution is highly skewed to the left [25,31]. HbA1c was considered as a dichotomous predictor (<39 mmol·mol^−1^ versus ≥39 mmol·mol^−1^) for the 25th, 50th and 75th percentiles of DAH30. Age, sex, BMI, ASA (1 or 2 versus ≥3), cardiovascular comorbidities (hypertension, ischaemic heart disease, stroke and peripheral vascular disease) and surgical risk (minor versus moderate/major risk) were used as covariates in both regression models. A *p*-value < 0.05 was deemed statistically significant.

## 3. Results

**Figure 1:**
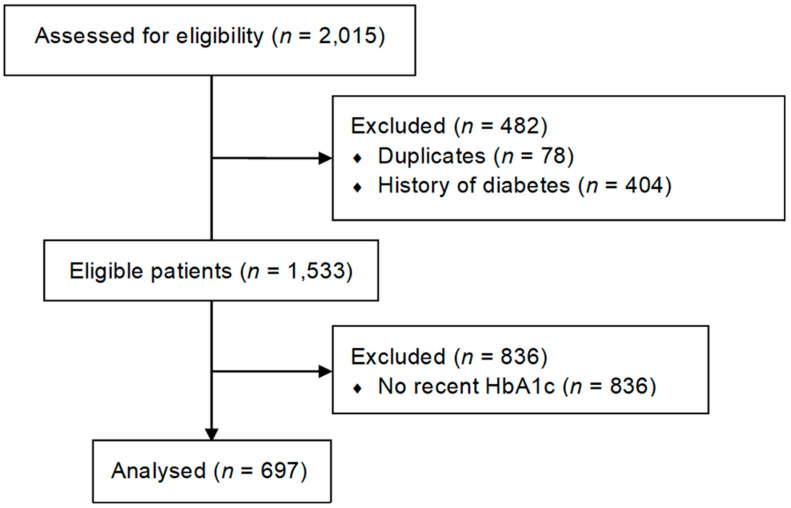
CONSORT diagram of patient recruitment.

From January to December 2019, 2015 patients aged ≥65 years underwent preoperative screening for elective non-cardiac surgery at our hospital. Duplicates were removed (*n* = 78) and patients with a diagnosis of DM (*n* = 404) were excluded, leaving 1533 patients without a history of DM. Of these, 697 had undergone HbA1c testing within 3 months before surgery (Figure 1). Demographic, clinical and surgical characteristics are shown in Table 1. Compared to patients without a recent HbA1c, the group of patients with an HbA1c within 3 months before surgery contained fewer patients with an ASA score of ≥3 and fewer patients undergoing major surgery (Table S1).

The mean preoperative HbA1c was 38.6 (±4.9) mmol·mol^−1^. We identified 26 cases of undiagnosed DM based on HbA1c, which corresponds to a prevalence of 3.7% (95%CI 2.5–5.4%) and translates into 27 as the number of patients needed to screen in order to detect one patient with undiagnosed DM. There were 299 subjects with a preoperative HbA1c in the prediabetes range, amounting to a prevalence of 42.9% (95%CI 39.2–46.7%) (Table 2).

Age (OR 1.05 [95%CI 1.02–1.08] per year; *p* = 0.001) was the only predictor of elevated HbA1c (i.e., ≥39 mmol·mol^−1^) to reach statistical significance in the multivariate regression model. The median (IQR) DAH30 was 29 (26–30) days in subjects with an HbA1c < 39 mmol·mol^−1^, 29 (26–29) days in subjects with an HbA1c in the prediabetes range and 29 (28–30) days in patients with undiagnosed DM based on HbA1c. In the multivariate regression model, preoperative HbA1c was not associated with DAH30 in the 25th, 50th and 75th percentiles (*p* = 0.88, *p* = 0.60 and *p* = 0.45, respectively).

## 4. Discussion

Based on epidemiologic data, we hypothesised that the routine measurement of HbA1c would lead to the discovery of undiagnosed DM in patients aged ≥65 years visiting our preoperative screening clinic. However, we found a low prevalence of undiagnosed DM (3.7%) in this older adult surgical population. Remarkably, 42.9% of patients without a diagnosis of DM had an HbA1c value within the prediabetes range. Other than age, we could not identify any risk factors for elevated HbA1c. We did not detect an association between HbA1c and DAH30 in this study population. 

In all cases of DM among the general population, the International Diabetes Federation (IDF) has estimated that 45% are undiagnosed in Europe [7]. One would expect the prevalence of undiagnosed DM in an older adult surgical population to be higher than the prevalence we found in our study population. However, a considerable number of patients seen at the preoperative screening clinic may have already undergone previous blood tests, potentially reducing the prevalence of undiagnosed DM among our surgical population compared to the general population. Several studies reported on the prevalence of undiagnosed DM in patients who planned to undergo surgery based on an HbA1c ≥ 48 mmol·mol^−1^. The majority of these studies included patients undergoing a specific type of surgery [32,33,34,35,36,37,38,39,40,41], such as cardiac [32,33,34,35], orthopaedic [36,37] and bariatric surgery [38,39]. In a prospective study conducted in a university hospital in Canada, as much as 7% of patients aged 18 years or older undergoing elective non-cardiac surgery were found to have undiagnosed DM based on their preoperative HbA1c value [27]. This difference in prevalence may be explained by their exclusion of ambulatory surgery, the relatively high number of patients classified as ASA 3–4 and geographical differences. Data from two other observational studies suggest a prevalence of undiagnosed DM similar to the prevalence in our study sample, i.e., 3.9% and 4.3%, despite the broader age range among participants in these studies [2,28]. Although the overall prevalence of DM increases considerably in each incremental age group [23], the proportion of diagnosed DM may be higher in older adults than in younger age groups. 

Although the diagnosis of DM is based on either the plasma glucose or HbA1c criteria [12,13,14], the measurement of the HbA1c value is likely the most convenient option in the preoperative assessment clinic, as it does not require fasting and is not affected by factors such as timing, stress or diet. Nonetheless, it is important to note that there is considerable discordance in the diagnosis of DM when comparing the HbA1c and plasma glucose criteria, as these tests measure different aspects of glucose metabolism [33,42]. An HbA1c above the diagnostic threshold may only detect one-third of the cases of undiagnosed DM that would otherwise be identified based on FPG and OGTT testing [43]. This may at least partly explain why the prevalence of undiagnosed DM that we found was lower than we expected based on previous population estimates. 

The association between preoperative HbA1c and postoperative outcome, specifically in the surgical patients without a diagnosis of DM, has been examined in several previous studies. Some studies, although not all [35,39,44], have found an association between HbA1c and various postoperative complications [9,45,46,47,48,49], length of hospital stay [50] and even mortality [46] (Table S2). It has been proposed that the risk of certain postoperative complications may be higher in patients with undiagnosed DM compared to patients with a history of DM [9,10,11]. In a retrospective cohort study that included patients undergoing peripheral arterial revascularization, subjects without a diagnosis of DM and an HbA1c above 53 mmol·mol^−1^ were found to be at a higher risk of amputation and adverse limb events compared to subjects with known DM and a similar HbA1c [9]. In another retrospective analysis, patients with undiagnosed DM were found to have a higher one-year mortality rate after non-cardiac surgery compared to patients with known DM [10]. However, FPG was used besides HbA1c to identify patients with undiagnosed DM in this study [10]. Similar observations have been made in critically ill patients with undiagnosed DM, who seem to have higher rates of dysglycaemia and increased mortality compared to patients with a diagnosis of DM [11]. We found no predictive value of HbA1c in relation to DAH30. 

Prediabetes is a heterogeneous condition characterised by glucose values or an HbA1c above the reference interval, yet below the DM diagnostic threshold. The criteria to diagnose prediabetes proposed by major international organisations are not uniform. The ADA and the National Institute for Health and Care Excellence guidelines have incorporated HbA1c as a means of diagnosing prediabetes [12,51], whereas the IDF and the World Health Organization define prediabetes based on glucose criteria only [23,52]. Prediabetes can progress to type 2 DM. However, prediabetes has no known relevance for the perioperative outcome. This raises the question of whether preoperative screening by HbA1c would contribute to public health, or if one should leave this to primary health care providers. Based on our data, in the Netherlands, the costs of detecting one patient with undiagnosed DM at the preoperative assessment clinic would be around GBP 255 (EUR 300), while it is unknown whether doing so would prevent any potential complications. However, there is some evidence that screening for (pre)diabetes could be effective in terms of long-term health benefits and cost-effectiveness [53].

The major strength of this study is its specific focus on older adult patients undergoing a diverse range of surgical interventions. However, some limitations in the study design should be noted. First, the exclusion of patients with a diagnosis of DM was solely based on the information from their electronic health records, though these records have proven to be reliable in earlier studies [54]. Second, although we implemented routine age-dependent screening of HbA1c in our preoperative assessment clinic, the majority of the patients who satisfied the criteria were not tested, which limited the sample size and may have introduced selection bias by excluding patients who were too ill to attend preoperative assessment or, conversely, were relatively healthy and were thus screened by telephone. Considering that the patients who were screened for HbA1c had a relatively lower ASA score and underwent fewer major surgical procedures compared to those who did not undergo HbA1c screening, we might have underestimated the prevalence of prediabetes and diabetes. Third, although we analysed the association between HbA1c and DAH30, the study was not powered to detect differences in this postoperative outcome. Finally, DAH30 was exclusively based on the hospitalisation data from our centre. Nonetheless, it is unlikely that post-discharge readmissions at other care facilities would have significantly influenced the study outcome, as postoperative patients will be referred to the initial clinic in case of postoperative complications within 30 days.

## 5. Conclusions

The prevalence of undiagnosed DM in older adult surgical patients is low. Although HbA1c screening revealed a large number of older adult patients with prediabetes, the relevance of this finding in the perioperative setting is uncertain. Considering the burden of DM in the perioperative setting, further studies are needed to identify the groups of surgical patients most at risk for undiagnosed DM. In these high-risk groups, glucose testing may be added to HbA1c screening alone to increase the DM detection rate. Based on our findings and the previous literature, general screening for HbA1c in older adult patients undergoing non-cardiac surgery appears to be of little value.

## Figures and Tables

**Table 1:** Demographic, clinical and surgical characteristics.

Characteristic	All Patients (*n* = 697)	No DM (*n* = 372)	Prediabetes (*n* = 299)	Undiagnosed DM (*n* = 26)
Age, years	73 (6)	72 (5)	73 (6)	76 (7)
Sex, female, *n*	323 (46.3%)	169 (45.4%)	143 (47.8%)	11 (42.3%)
BMI, kg m^−2^	26 (4)	26 (4)	26 (4)	27 (4)
ASA score, *n*				
1 or 2	473 (67.9%)	260 (69.9%)	197 (65.9%)	16 (61.5%)
≥3	224 (32.1%)	112 (30.1%)	102 (34.1%)	10 (38.5%)
Maximum MET, *n*				
<7	240 (39.5%)	126 (38.8%)	104 (39.8%)	10 (47.6%)
≥7	367 (60.5%)	199 (61.2%)	157 (60.2%)	11 (52.4%)
Cardiovascular history, *n*				
HT	317 (45.5%)	160 (43.0%)	141 (47.2%)	16 (61.5%)
IHD	96 (13.8%)	40 (10.8%)	50 (16.7%)	6 (23.1%)
CVA/TIA	76 (10.9%)	43 (11.6%)	29 (9.7%)	4 (15.4%)
PVD	25 (3.6%)	11 (3.0%)	14 (4.7%)	0
Surgical specialty, *n*				
Gynaecology	49 (7.0%)	26 (7.0%)	22 (7.4%)	1 (3.8%)
Gastrointestinal	95 (13.6%)	54 (14.5%)	38 (12.7%)	3 (11.5%)
Orthopaedic	106 (15.2%)	62 (16.7%)	40 (13.4%)	4 (15.4%)
Urology	62 (8.9%)	36 (9.7%)	23 (7.7%)	3 (11.5%)
Neurosurgery	37 (5.3%)	19 (5.1%)	16 (5.4%)	2 (7.7%)
Other	349 (49.9%)	175 (47.0%)	160 (53.5%)	13 (50.0%)
Surgical risk, *n*				
Minor	418 (60.0%)	224 (60.2%)	177 (59.2%)	17 (65.4%)
Moderate	220 (31.6%)	121 (32.5%)	92 (30.8%)	7 (26.9%)
Major	59 (8.5%)	27 (7.3%)	30 (10.0%)	2 (7.7%)
Anaesthesia type, *n*				
General	594 (85.3%)	319 (85.8%)	253 (84.6%)	22 (88.0%)
Neuraxial	36 (5.2%)	20 (5.4%)	15 (5.0%)	1 (4.0%)
PNB	24 (3.4%)	15 (4.0%)	9 (3.0%)	0
Sedation	42 (6.0%)	18 (4.9%)	22 (7.4%)	2 (8.0%)

Values are number (proportion) or mean (SD). HT, hypertension; IHD, ischemic heart disease; CVA/TIA, cerebrovascular accident and/or transient ischaemic attack; MET, metabolic equivalent of task; PNB, peripheral nerve block; PVD, peripheral vascular disease.

**Table 2:** Prevalence of undiagnosed diabetes and prediabetes.

	HbA1c (mmol mol^−1^)	HbA1c (%)	*n*	Prevalence (%)	95% Confidence Interval
No diabetes	<39	<5.7%	372	53.4	49.6 to 57.1
Prediabetes	≥39 and <48	5.7–6.5%	299	42.9	39.2 to 46.7
Diabetes	≥48	6.5%	26	3.7	2.5 to 5.4

## Data Availability

The raw data supporting the conclusions of this article will be made available by the authors on request.

## Supplementary Materials

The following supporting information can be downloaded at: https://www.mdpi.com/article/10.3390/jpm14020219/s1, Table S1: Comparison of demographic, clinical and surgical characteristics between patients with and without a recent HbA1c; Table S2: Other observational studies reporting on the association between HbA1c and postoperative outcome in (a subgroup of) patients without a diagnosis of diabetes.
