# Supplementary material for: Preoperative Glycosylated Haemoglobin Screening to Identify Older Adult Patients with Undiagnosed Diabetes Mellitus—A Retrospective Cohort Study
Source: J Pers Med. 2024 Feb 19;14(2):219. doi: 10.3390/jpm14020219 (PMC10890067; doi:10.3390/jpm14020219)
Supplement: Supplementary file 1 [file jpm-14-00219-s001.zip › jpm-2861255-supplementary.pdf]

Supplementary tables

Table S1: Comparison of demographic, clinical and surgical characteristics between patients with and without a recent HbA1c.

Characteristic	**No recent HbA1c (n = 836)**	**Recent HbA1c (n = 697)**	**p**
**Age, years**	73 (6)	73 (6)	0.027
**Sex, female, n**	479 (57.3%)	323 (46.3%)	< 0.001
**BMI, kg m^{-2}**	26 (5)	26 (4)	0.048
**ASA score, n**			
**1 or 2**	496 (59.3%)	473 (67.9%)	< 0.001
**≥ 3**	340 (40.7%)	224 (32.1%)	< 0.001
**Maximum MET, n**			
**< 7**	327 (44.7%)	240 (39.5%)	0.059
**≥ 7**	405 (55.3%)	367 (60.5%)	0.101
**Cardiovascular history, n**			
**HT**	363 (43.4%)	317 (45.5%)	0.42
**IHD**	136 (16.3%)	96 (13.8%)	0.18
**CVA/TIA**	84 (10.0%)	76 (10.9%)	0.59
**PVD**	26 (3.1%)	25 (3.6%)	0.60
**Surgical specialty, n**			
**Gynaecology**	171 (20.5%)	49 (7.0%)	< 0.001
**Gastrointestinal**	121 (14.5%)	95 (13.6%)	0.636
**Orthopaedic**	52 (6.2%)	106 (15.2%)	< 0.001
**Urology**	77 (9.2%)	62 (8.9%)	0.831
**Neurosurgery**	71 (8.5%)	37 (5.3%)	0.015
**Other**	344 (41.1%)	349 (49.9%)	< 0.001
**Surgical risk, n**			
**Minor**	421 (50.4%)	418 (60.0%)	< 0.001
**Moderate**	275 (32.9%)	220 (31.6%)	0.579
**Major**	140 (16.7%)	59 (8.5%)	< 0.001
**Anaesthesia type, n**			
**General**	696 (83.4%)	594 (85.3%)	0.293
**Neuraxial**	23 (2.8%)	36 (5.2%)	0.014
**PNB**	14 (1.7%)	24 (3.4%)	0.027
**Sedation**	102 (12.2%)	42 (6.0%)	< 0.001

Values are number (proportion) or mean (SD). HT, hypertension; IHD, ischemic heart disease; CVA/TIA, cerebrovascular accident and/or transient ischaemic attack; MET, metabolic equivalent of task; PNB, peripheral nerve block; PVD, peripheral vascular disease.

Table S2: Other observational studies reporting on the association between HbA1c and postoperative outcome in (a subgroup of) patients without a diagnosis of diabetes.

	Type	Population*	Postoperative outcome
**Gustafsson (2009)**	Prospective	N = 120<br>Age: 31-90<br>Colorectal surgery	HbA1c > 43 was associated with a higher rate of 30-day overall complications (OR 2.9 [95%CI 1.1-7.9]; p = 0.037)
**Walid (2010)**	Retrospective	N = 383 (442)<br>Age: 21-92<br>Spine surgery	HbA1c ≥ 43 was associated with increased LOS (7.0 (5.9) vs 4.7 (2.5) days; p < 0.05) and higher total cost in 1 of 3 subgroups (lumbar decompression and fusion).
**Hudson (2010)**	Retrospective	N = 1615<br>Age: 65 (11)<br>Cardiac surgery	Increased 30-day mortality (OR 1.53 per 11 $mmol.mol^{-1}$ HbA1c increase [95%CI 1.24-1.91]; p < 0.001); HbA1c > 43 was associated with an increased rate of AKI (OR 1.148 [95%CI 1.003-1.313]; p = 0.04). No association between HbA1c and infection.
**Iavazzo (2015)**	Prospective	N = 266<br>Age: unknown<br>Gynaecologic surgery	No association between HbA1c and rate of complications or 30-day readmissions
**Gatti (2017)**	Prospective<br>(E-CABG registry)	N = 1384<br>Age: 68 (9)<br>Cardiac surgery	HbA1c > 44 was an independent predictor of sternal wound infections (OR 2.23 [95%CI 1.17-4.2]; p = 0.012)
**Narayan (2017)**	Retrospective	N = 1633<br>Age: 59 (9)<br>Cardiac surgery	No association between HbA1c and complications or 30-day mortality
**Nicolini (2018)**	Prospective<br>(E-CABG registry)	N = 1664<br>Age: 68 (9)<br>Cardiac surgery	HbA1c > 53 was associated with a higher rate of stroke (adjusted OR 5.68 [95%CI 1.74-18.57]; p < 0.001) and prolonged inotropic support (adjusted OR 1.88 [95%CI 1.17-3.03]; p = 0.01)
**Kocogullari (2018)**	Retrospective	N = 202<br>Age: 33-82<br>Cardiac surgery	HbA1c ≥ 38 was associated with a higher rate of AKI (adjusted OR 11.17 [95%CI 2.21-56.33]; p = 0.003)
**Arya (2018)**	Retrospective<br>(Veterans Health Administration database)	N = 10,746<br>Age: 66 (9)<br>Vascular surgery	Higher rate of amputation (HR 1.49 [95%CI 1.06-2.11] for HbA1c 54-64 vs. < 42; HR 1.98 [95%CI 1.51-2.59] for HbA1c > 64 vs. < 42). Higher rate of modified major adverse limb events (HR 1.19 [95%CI 0.95-1.48] for HbA1c

			54-64 vs. < 42; HR 1.52 [95%CI 1.25-1.84] for HbA1c > 64 vs. < 42)
**Wysocki (2019)**	Retrospective	N = 1375<br>Age: 41 (35-51)<br>Bariatric surgery	No association between HbA1c and postoperative morbidity, prolonged LOS or hospital readmission
**Lam (2022)**	Prospective (UK Biobank cohort study)	N = 26,653<br>Age: 58 (8) (HbA1c < 42)<br>61 (7) (HbA1c ≥ 42)<br>Cardiac and non-cardiac surgery	HbA1c ≥ 42 was associated with a higher rate of the composite endpoint of 30-day major postoperative complications and 90-day all-cause mortality

Values are mean (SD) or median (Q1-Q3).

* If the study sample also included patients with a diagnosis of diabetes, the total sample size is shown between brackets. Only the outcome data of patients without a history of diabetes are shown.

AKI, Acute Kidney Injury; CABG, Coronary Artery Bypass Grafting; CPB, cardiopulmonary bypass; PAD, peripheral arterial disease; HbA1c, glycosylated haemoglobin ($mmol.mol^{-1}$).
